# Rheumatic symptoms in childhood leukaemia and lymphoma-a ten-year retrospective study

**DOI:** 10.1186/1546-0096-11-20

**Published:** 2013-05-04

**Authors:** Luca Zombori, Gabor Kovacs, Monika Csoka, Beata Derfalvi

**Affiliations:** 1 2nd Department of Paediatrics, Semmelweis University Budapest, 1094, Tuzolto u 7-9, Budapest, Hungary

**Keywords:** Childhood, Musculoskeletal complaints, Leukaemia, Lymphoma, Juvenile, Idiopathic arthritis

## Abstract

**Background:**

The initial symptoms of childhood leukaemia and lymphoma are often similar to those of juvenile idiopathic arthritis (JIA). In our study, we analyzed the frequency and characteristics of musculoskeletal complaints as the initial presenting symptoms of newly diagnosed leukaemia and lymphoma patients in the past 10 years in our clinic.

**Methods:**

Using the Hungarian Tumour Register, we performed a retrospective analysis of the medical records of 166 new leukaemia and 95 new lymphoma pediatric patients treated from 1999 to 2009 at the 2nd. Dept. of Paediatrics of the Semmelweis University in Budapest.

**Results:**

Twenty percent of the leukaemic (33 children) and 2% of the lymphoma patients (2 children) had musculoskeletal symptoms at first presentation. Two-thirds of both groups of patients had other general symptoms like fever and/or fatigue. The hip was the most frequently affected joint (7/33) in the leukaemic patients. Twenty-four percent of all the children had been previously evaluated by an orthopaedist; 12% had visited another rheumatologist prior to diagnosis. Imaging had been done in an unexpectedly low number of patients prior to referral to our unit (radiographs: 16 or 48%, ultrasound: 5 patients or 15%). Radiographs of the affected joints were abnormal in only one case (1/16, 6%). The joint ultrasound was abnormal in only three children of 5 studied (3/5, 60%). Anaemia (26/32, 6%), thrombocytopenia (78%) and LDH elevation (3–4 times the normal count) were frequent in the leukaemic patients. Half of the cases had a normal leukocyte count. The lymphoma group had similar results. Two patients of the leukaemia group received steroid treatment before the final diagnosis. Severe pain out of proportion to physical findings is another clue.

**Conclusions:**

Haematologic malignancies must be excluded before initiation of therapy for childhood arthritis among children presenting with musculoskeletal signs and symptoms, particularly in atypical cases. Malignancies are to be suspected when pain is disproportionately severe compared to the physical examination findings, and when anaemia, thrombocytopenia, and an elevated LDH level are present. Diagnosing leukaemia early is important because the use of steroids and immunosuppressive medications may mask and delay its diagnosis. Additionally, pre-treatment of presumed JIA patients with these drugs who eventually are diagnosed to have a malignancy may lead to the malignancy being steroid-resistant and more difficult to treat.

## Background

Transient joint and limb pain is common among children, and in the majority of cases resolves without any treatment. These pains are mostly caused by benign growing pains or hypermobile joints. It is unusual that these pain complaints prove to be of organic origin, and even more unusual that a malignancy is the cause. One percent of paediatric musculoskeletal complaints are caused by neoplasia, most frequently acute lymphoblastic leukaemia (ALL) [1,2]. Acute lymphoblastic leukaemia is the most common childhood malignant disease, comprising 25% of all paediatric malignancies [3].

ALL predominantly affects children between 1 and 5 years of age with a male to female ratio of 1.3:1. The onset of the disease can be lengthy with a prodromal stage lasting for weeks, or even months. Initial symptoms are often non-specific, such as fever, fatigue, pallor, or limb and joint pain. Laboratory variables initially are also often nonspecific; e.g., the white blood cell count is normal in half the cases of children presenting with leukaemia [4].

Childhood rheumatic inflammatory diseases, such as juvenile idiopathic arthritis (JIA), may begin with similar general symptoms, especially the joint complaints. Unfortunately, in children with the musculoskeletal presentation of leukaemia and lymphoma, lab tests and imaging at first may not detect malignancy, and repeated testing and prolonged observation and follow-up over weeks may be needed to diagnose the malignancy [1-4].

After our experiences of diagnosing children with malignancies in our clinic, we began this study to determine how prevalent musculoskeletal complaints are in the Hungarian population of leukaemia and lymphoma patients.

## Methods

By utilizing the national database of the Hungarian Paediatric Tumour Register, we conducted a retrospective institutional study at the 2nd. Dept. of the Semmelweis University of Budapest of all 166 new leukaemic and 95 new lymphoma patients treated from 1999 till 2009. All subject’s medical records of children treated with a haematologic malignancy were analyzed. The data of the first visit to the haematogist-oncologist at the time of the diagnosis was reviewed by the rheumatologist. The data collection and analysis has been approved by the Institutional Review Board of the University.

### Aims

Our questions were the following: What percent of the cases diagnosed as hematologic malignancies in our population start with a rheumatic or musculoskeletal complaint? Are there similarities or differences regarding the location of the complaint, pattern, time of the first presenting symptom, or the laboratory and radiologic findings compared to that of juvenile idiopathic arthritis? When do we have to suspect a haematologic malignancy in a child presenting with musculoskeletal complaints?

### Definitions

a) Musculoskeletal/rheumatic complaints: Arthralgia, arthritis, bone pain, arthromyalgia, inability to walk

b) Arthralgia: joint pain without the objective signs of inflammation such as swelling, warmth with/or without restricted range of motion

c) Arthritis: Joint pain with signs of inflammation or synovitis with or without joint pain.

d) Bone pain: Pain not localized to a joint.

e) Arthromyalgia: bone and muscle pain present at the same time.

f) Oligoarthritis: Arthritis affecting less than 5 joints,

g) Polyarthritis: Arthritis affecting 5 or more joints.

h) Lymphadenopathy: A lymph node with a diameter greater than 1 centimetre.

a. Hepatomegaly: A liver exceeding the rib cage by more than one finger`s width,

i) Splenomegaly: A spleen exceeding the rib cage by more than one finger`s width.

j) Anaemia: Haemoglobin concentration under 100 g/l,

k) Thrombocytopenia: A platelet count. under 150 G/l

l) Low-normal platelet count: The range between the values of 150–250 G/l

m) Leukocytosis: WBC values more than 14.3 G/l,

n) Leukopenia: WBC values fewer than 3.8 G/l.

o) LDH count: LDH values greater than 400 U/l was considered abnormal

p) CRP: Less than 20 mg/l.

q) Hyperuricaemia: Greater than 420 umol/l.

Note: The laboratory results were collected at the first presentation of our clinic, at an average of 10 days after the onset of the first symptoms.

## Results

### Leukaemia

We analysed the data of the 166 newly diagnosed leukaemic patients during the above defined period of time. One hundred and twenty-four (75%) of the children were male. The median age at the diagnosis was 5.1 years (range: 1.4-16.4 years). One hundred and sixty-one (97%) of the children had acute lymphoblastic leukaemia (ALL) as the final diagnosis and 3% acute myeloid leukaemia (AML). We found musculoskeletal symptoms as the first presentation of the disease in 20% of the children with leukaemia (33/166) and in only 2% (2/95) of the children with lymphoma. We next did the analysis of this group focusing on the children with leukaemia or lymphoma with a musculoskeletal presenting symptom and noting the location and character of the symptoms.

Two-thirds (22/33) of the leukaemic children with musculoskeletal symptoms had other symptoms beside musculoskeletal complaints, most commonly fever, irritability or fatigue. Other associated symptoms and their frequency are shown in Table 1.

**Table 1:** Clinical symptoms in ALL pediatric patients presenting with musculoskeletal complaints

**Symptom**	**Frequency**	**Percentage**
Fever	10/33	30%
Slight prolonged hyperthermia	3/33	9%
Irritability	8/33	24%
Fatigue	7/33	21%
Paleness	3/33	9%
Abdominal pain	3/33	9%
Swollen face	2/33	6%
Vomiting	2/33	6%
Dizziness	1/33	3%

Musculoskeletal or rheumatologic symptoms included arthromyalgia in about half of the group (54%, 18/33), followed by arthralgia (36%, 12/33) and arthritis (26%, 8/33). Bone pain was less frequent (12%, 4/33), but significant as it is not typical for JIA. An limp was present in 15% (5/33), and another 9% became unable to walk (3/33). On physical examination, the joints were less abnormal than expected, and in the presence of arthritis, inflammatory parameters were also not as high, and not as abnormal as might be predicted from the pain severity. Arthralgia and arthritis counted together were monoarticular in involvement in 45% (15/33), as well as oligo/pauciarticular involvement in 55% (18/33) of the cases. Sex and age distribution of children with arthralgia or arthritis were the following: 79% (26/33) male, (average age 7.2 years), 21% (7/33) female (average age 5.1 years). Mono/oligoarticular arthritis was present in 6 boys and 2 girls. There was no child with polyarticular arthritis in our study. The hip was the most frequently affected (7/33) joint, followed by the knee and the back (Table 2).

**Table 2:** Joint involvement in pediatric ALL patients presenting with arthritis and/or arthralgia as the first complaints

**Joint**	**Frequency**	**Percentage**
Hip	7/33	21%
Knee	3/33	9%
Spine	3/33	9%
Tarsal	2/33	6%
Lumbal	2/33	6%
Shoulder	1/33	3%
Ankle	1/33	3%
Limbs	1/33	3%
Diffuse pain	4/33	12%

In the initial stages of the illness, 76% of the children with leukaemia who experienced rheumatologic symptoms first presented at their family practitioner; 24% sought an orthopaedist, and 12% a rheumatologist. Final diagnoses occurred at a clinic or hospital in 85% of the cases; 9% were made by the family practitioner, and 3.3% by an orthopaedist or rheumatologist.

Eighty-six percent of the leukaemic children had anaemia (26/33) and 75% had thrombocytopenia. The children with thrombocytopenia plus the children with low-normal platelet count totaled 91% of the 33 leukaemic children. For the white blood cell count, half of the cases had a normal value at the first measure, 39% had leukocytosis and 9.6% had leucopenia. Ninety-six percent of the ALL patients had absolute or relative lymphocytosis. These results are compatible with previous reports and support that the diagnosis of leukemia often cannot be initially established based on the peripheral blood counts alone. Repeat testing of the complete blood count are needed over the next few days to weeks for abnormalities to emerge [5-7].

The LDH was elevated in 97% (32/33). A two-fold increase of the normal level was present in 48% (16/33), and a three-fold increase in 33% of the cases (11/33). The CRP exceeded the normal range in 80% of the cases. The uric acid was normal in most cases as hyperuricaemia was seen in only 8%.

Radiological examination abnormalities were rarely seen at the first presentation of symptoms. A radiograph of the affected joint or limb was performed in about half of the cases (16/33); only one was abnormal with osteolytic lesions on the os ischii. Ultrasound was used in 5 cases, (5/33), and 3 showed inflammation of the joint with the presence of fluid. Three children had a musculoskeletal MRI, but only one was abnormal.

The average time lapse between the onset of symptoms and diagnosis of leukemia was 52 days, ranging from 5 to 721 days. This included two children with very prolonged periods prior to diagnosis, almost three years in one, and 9 months in another.

The first diagnosis in these two cases was JIA. It is also possible, of course, that the child experienced two illnesses, one after another: first JIA, then a leukaemia, manifesting itself later. It is more likely that since such a lengthy prodrome is not typical for leukaemia, the temporary amelioration of symptoms was the result of the JIA therapy. Therapy for the children with erroneous JIA diagnoses were NSAID’s in 12% of the cases while two children (both in the leukaemia group) received steroid therapy (6%, 2/33). All of the above therapies proved to be temporarily useful for the alleviation of the pain symptoms, and leukaemia was only diagnosed after recurrence of the chronic pain.

### Lymphoma

In the 95 children diagnosed with lymphoma, only 2 cases initially presented with a rheumatologic symptom. In the first case a 17-year-old boy complained of left sided hip pain present for 3 months. A hip radiograph suggested a lytic lesion. A hip MRI described a pathological bone lesion in the affected region. A biopsy was performed, proving the diagnosis of a lymphoma. The second case involved a 9-year-old girl who complained of thigh pain and intermittent fever for 6 months. Her symptoms were alleviated by non-steroid anti-inflammatory drugs (NSAID). An elevated CRP, LDH and ferritin levels were noted. Bone scintigraphy was performed and suggested Paget syndrome. MRI described patches of bone lesions and the resulting biopsy gave the final diagnosis of lymphoma.

## Discussion

Haematological malignancies must be ruled out in children with musculoskeletal complaints. This is especially true if the pain is consistently strong, wakes the child at night, or localizes to a non-joint area, as these features are not characteristic for JIA, the most frequent chronic childhood rheumatologic disease [1]. The frequent, so-called growing pains, are never accompanied by a laboratory or radiological alteration.

What causes the musculoskeletal complaints in a leukaemia or lymphoma? Infiltration of the synovia, the periosteum, or the bone marrow by malignant cells is one explanation, but a secondary uric acid or immune complex deposits may also cause pain and inflammation in a joint. A less common factor may be an intraarticular bleeding resulting from thrombocytopenia [4].

Our results support previous reports that suggest that pain disproportionate to physical findings, accompanied by haematologic alterations and pain not permanently alleviated by NSAIDs, strongly suggest the presence of a haematologic malignancy [5,8]. But in contrast to other reports, only 20% of the newly diagnosed ALL patients had musculoskeletal symptoms, one third of them with accompanying general complaints. Fever, pallor, bone pain and night pain are the most likely symptoms of an underlying leukaemia [9,10]. Severe night-time pains that awaken a child are not typical for JIA and point towards malignant diseases [8].

In most of our cases, arthralgias were localised at the hip joint. Other authors, e.g., Barbosa et al. found that rheumatologic pain of leukemic origin may also affect the lower limbs, especially the knee [11]. Other reports mention wrist, ankle, and elbow involvement as well [8]. In contrast, Trapani et al. found arthritis as the most frequent presenting symptom, mostly affecting one large joint in ALL [2]. Arthralgia was less frequent in their study, localising mostly at the shoulders, arms, spine and knee. Arthritis was almost never accompanied by morning joint stiffness. Stiffness of the joints is an important symptom suggesting JIA over a malignancy. Interestingly, 79% of leukaemia patients in our series presenting with mono/oligo arthritis or arthralgia were boys. Thus in younger male children with oligoarticular disease the possibility of leukemia should also be entertained, especially because the 2/3 of oligoarticular JIA patients are girls. Lymphadenopathy and hepatosplenomegaly can be present in both ALL and JIA, so we don’t regard it as a good differential diagnostic feature.

Regarding the laboratory testing, we would like to emphasize the presence of thrombocytopenia in the leukemic patients and point out that juvenile idiopathic arthritis presents more often with thrombocytosis, which results from the stimulation of megakaryocytes by IL-6 as part of the acute phase reaction [12]. Barbosa et al. emphasize the importance of the elevated LDH level [11], with the neoplasia group having a 2.2 times the normal range elevation, while the JIA group only a 0.8 times normal. Our study found that LDH in the neoplasia group was usually 3–4 times above the normal range. Another report suggested that the LDH was also a good marker for therapy and prognosis and of a malignant disease [2].

Most of the patients were anaemic in an Indian publication similar to our findings, although anaemia in itself is not always that typical for leukaemia [6,10]. The white blood cell count can be normal, elevated of decreased at the beginning of the ALL, but absolute or relative lymphocytosis is very characteristic to ALL.

ANA positivity is common in the oligoarticular, psoriasis-associated and polyarticular JIA, but it doesn’t exclude the presence of haematologic malignancies. Strong ANA positivity has been found in 17% of children with ALL and 5% of children with diverse lymphoproliferative diseases [6,13].

The importance of x-rays in early detection of leukaemia has been emphasized by several authors. Emphasis is placed on boney signs present in some children with leukaemia, such as osteoporosis, raindrop-like lytic lesions, radiolucent metaphyseal bands on long bones, and wedge fractures of vertebrae [7]. Radiological signs of ALL are seen in 45-70% of children at presentation, although these lesions may not be easy to detect [14]. This high rate was not typical of our study group. We would like to note, however, that an x-rays was performed in a far fewer number of our cases than performed in some other reports in the literature.

Marwaha and his colleagues suggest that ALL patients whose disease begins with rheumatologic symptoms have a better 5-year survival rate then non-rheumatologic ALL patients. In this rheumatologic symptom group, an eventual relapse also usually starts with these musculoskeletal symptoms. Thus this group may be categorized as a subgroup of ALL in the future [10].

Two of the children received steroid treatment when the initial suspected diagnosis of JIA was made. The danger of steroid treatment is that it may mask the symptoms, signs, and abnormal laboratory tests of leukaemia; the correct diagnosis and immuncytologic typing might be delayed and disturbed. Finally, it may may lead to reclassification of the disease to a higher risk group, according to the IC BFM 2002 protocol for ALL used in our institute [15,16]. While survival in the low risk group is 80-90%, that of the high risk group is only 60%, resulting not only from the stage or progression of the leukaemia, but also from side effects of the more aggressive treatment.

## Conclusions

Awareness of the possibility of a haematologic malignancy, such as leukaemia, in a child presenting with musculoskeletal complaints is essential. This is especially important when the signs and complaints are not typical of the paediatric rheumatologic and musculoskeletal diseases and syndromes, e.g. juvenile idiopathic arthritis, growing pains and hypermobile joints. The most frequent signs of malignancies presenting with a musculoskeletal presentation are migratory bone and joint pain with variable intensity and limping without significant physical findings. These children often are febrile and have elevated inflammatory laboratory markers such as the ESR and CRP. In our patients, we found the following signs useful to suggest a haematologic malignancy in children experiencing musculoskeletal and rheumatologic symptoms: pain disproportionate to the physical findings, lack of morning stiffness typical of arthritis, lymphadenopathy, hepatosplenomegaly, thrombocytopenia, anaemia, and a LDH 2–5 times the normal range of LDH.

If malignancy is strongly suspected, a definitive diagnosis should be made by a bone marrow biopsy, as blasts are often not present on the peripheral smear of the CBC. Malignancy should be eliminated as a possibility before the introduction of steroid or immunosuppressive therapy for an arthritis disease.

## Competing interests

The authors have no financial competing interests to report.

## Authors’ contributions

ZL participated in the acquisition, analysis and interpretation of data, KG and CSM were involved in revision of the manuscript and were important, for the intellectual content of the manuscript. DB have made substantial contributions to conception and design, participated in coordination and helped to draft the manuscript. All authors read and approved the final manuscript.
